# Editorial: Research advances in mucormycosis

**DOI:** 10.3389/fcimb.2023.1280784

**Published:** 2023-09-06

**Authors:** Rachna Singh

**Affiliations:** Vallabhbhai Patel Chest Institute, University of Delhi, Chandigarh, India

**Keywords:** mucormycosis, Covid-19 associated mucormycosis, rhizopus, mucor, environment, diagnosis, treatment

Fungi belonging to the class Zygomycetes and the order *Mucorales* are opportunistic pathogens that cause acute, aggressive angio-invasive infections with high mortality rate. The infections are categorized into rhino-orbito-cerebral (ROC), pulmonary, gastrointestinal, cutaneous and disseminated forms, based on the clinical presentation and anatomical sites involved. Each of these types is associated with distinct risk factors. Significant differences have been noted in the epidemiology of mucormycosis in developed versus developing countries. The incidence of this disease has increased worldwide over the past few decades but the rise in developing countries, including India, has been phenomenal (Chakrabarti and Singh, 2014; Prakash and Chakrabarti, 2021). The situation was further aggravated by an upsurge of mucormycosis cases in the backdrop of Covid-19, with Covid-19 associated mucormycosis (CAM) being declared a notifiable disease (Rudramurthy et al., 2021; Hoenigl et al., 2022). This Research Topic aimed to explore the advances and innovations relevant to mucormycosis, focusing on diverse aspects of this disease, such as pathobiology, diagnostic modalities, prophylactic measures, and therapeutic interventions.

*Mucorales* are thermotolerant in nature and ubiquitous in their distribution, commonly occurring on organic substrates such as bread, decaying fruits and vegetables, crop debris, compost piles, animal excreta and soil in the outdoor and indoor environment. The disease is predominantly community acquired, and only 9% of the cases are nosocomial in origin (Chakrabarti and Singh, 2014; Prakash and Chakrabarti, 2021). Environment is considered a key component of the epidemiological triad of this disease, but its precise role was not much investigated during CAM. High mucoralean spore counts in the hospital and its vicinity have been reported not only during pandemic but also during pre-pandemic period in India (Biswal et al., 2022). The study by Ghosh et al. in the present Research Topic determined the role of environmental contamination from the patients’ residences as a source of infection. The authors evaluated the environmental spore counts in and around the residence of 25 patients who developed CAM while convalescing at home post-COVID-19, and observed that the median spore counts were significantly higher in the patients’ bedroom (3.55; range, 0 to 16 cfu/m^3^) compared to the other rooms (1.5; range, 0 to 7 cfu/m^3^) and water cooler (0; range 0 to 8 cfu/m^3^), and even outdoors (2, range 0 to 15 cfu/m^3^). In 68% of the cases, the same species of *Mucorales* was isolated from the clinical sample and the residence. Genetic relatedness between the clinical and the corresponding environmental isolate was observed in 44% of the cases based on amplified fragment length polymorphism. This correlates with the fact that the patients with Covid-19 were quarantined for a period of two weeks or more, with limited trafficking and cleaning, which may have aided fungal occurrence, sporulation and dispersal. The study suggests that the patients with high risk of acquiring mucormycosis should wear an appropriate mask even at home to minimize exposure. The authors also propose the use of portable air cleaners, heating, ventilation and air conditioning (HVAC) filters, indoor humidifiers, regular aeration of quarantine rooms and exposure to sunlight for minimizing spore counts and reducing spore dispersal in the homes of high-risk patients.

Owing to the rapid disease progression and fatality, early diagnosis of mucormycosis is critical for its management. However, it remains a major challenge. Conventional assays, including microscopy and culture, are relatively arduous due to the fragile nature of these fungi, low viable counts in the tissues and exudates, and distortion of fungal morphology/viability during processing. Also, microscopy and histopathology are not able to identify the fungus up to genus or species level, except the recently reported monoclonal antibody-based immunohistochemistry assay. Culture can be false negative in nearly 50% of the cases. Molecular techniques targeting 18S rDNA, 28S rDNA, internal transcribed spacer, high-affinity iron permease, spore coat protein Cot H, cytochrome b or mitochondrial large-subunit-ribosomal-RNA rnl have been tested in various formats; however, standardized and commercial assays are not much available (Cornely et al., 2019). Literature is also relatively silent on molecular assays for simultaneous detection of invasive fungal pathogens like *Aspergillus* spp. and *Mucorales.* In this regard, the study by Pandey et al. in the present Research Topic reports the development of a real time PCR assay for simultaneous detection and differentiation of *Aspergillus* spp. and *Mucorales* by melting curve analysis. The work utilizes a single set of primers which amplify a 222 bp (*Fusarium*) or 223 bp (*Aspergillus* and *Mucorales*) fragment in 18rDNA for identification up to genus level based on the melting temperature, which was found to be 83.5, 85.5 and 87°C for *Mucorales, Aspergillus* and *Fusarium* respectively. The assay was evaluated in site-specific samples and found to have a sensitivity and specificity of 99.29 and 83.84% respectively, for the diagnosis of invasive mucormycosis, and 93.3 and 97.1% respectively, for the diagnosis of invasive aspergillosis. The primer also amplified a larger fragment of dermatophytes (∼472 bp), with a melting temperature of 95°C, although it was not evaluated further. In yet another study by Aerts et al., the commercially available real time PCR assay, MucorGenius^®^, was used for evaluating the possibility of dual mould infections. The authors retrospectively tested *Mucorales* PCR on the banked sera samples from patients diagnosed with invasive aspergillosis. Serum *Mucorales* PCR was found to be positive in 8.7% of proven IA cases and 12.9% of probable IA compared with 0.5% of the control cases. However, there was only one unique sample positive for PCR and the consecutive samples collected before and after the diagnostic sample were negative, raising the possibility of false positivity, adequate treatment or spontaneous clearance. Further data is necessary to merit the value of this approach. Also, a prospective study would shed greater insights into its applicability for routine clinical practice.

With respect to the treatment options, intravenous administration of amphotericin B lipid derivatives remains the first line of therapy against mucoralean fungi. The newer azoles, posaconazole and itraconazole, administered intravenously, are moderately recommended as first lines of therapy *per se*, and strongly recommended in patients with pre-existing renal compromise. Based on the response assessment, the first line treatment can be continued or switched over to oral treatment with posaconazole or isavuconazole (Cornely et al., 2019). In the present Research Topic, Zhao et al. report successful treatment of a case of rhino orbito cerebral mucormycosis complicated with fungal endophthalmitis by administration of intravenous amphotericin B colloidal dispersion and oral posaconazole, along with intravitreal amphotericin B and surgical debridement. The case demonstrates the safety and efficacy of combination treatment and topical treatment.

In conclusion, progress has been made in the current Research Topic on understanding the role of environment as a contributory factor for CAM, and development of molecular method for early and simultaneous detection of the two key invasive mould infections (aspergillosis and mucormycosis). The research in mucormycosis is largely hindered by the fact that very few groups are working on the pathophysiology of this disease. Many lacunae still remain, such as evaluating the role of genetic susceptibility, host microbiome and immune dysfunction/dysregulation in the emergence of mucormycosis. Also, further research on novel treatment options, including newer and investigational antifungal drugs, and immunotherapies against CotH proteins is the need of the hour. It is well known that mucoralean fungi exhibit interspecies variation in terms of virulence, immune response and antifungal susceptibility. Cross-species validation is therefore critical, as majority of the available work focuses on *Rhizopus arrhizus*. It is imperative that the global attention this disease received due to the upsurge of CAM gets translated into greater R & D inputs and progress.

## Author contributions

RS: Conceptualization, Data curation, Formal Analysis, Investigation, Software, Validation, Writing – original draft, Writing – review & editing.
